# Effect of Chinese Patent Medicines on Ocular Fundus Signs and Vision in Calcium Dobesilate-Treated Persons With Non-Proliferative Diabetic Retinopathy: A Systematic Review and Meta-Analysis

**DOI:** 10.3389/fendo.2022.799337

**Published:** 2022-03-14

**Authors:** Yuehong Zhang, Xuedong An, Liyun Duan, De Jin, Yingying Duan, Rongrong Zhou, Yuqing Zhang, Xiaomin Kang, Fengmei Lian

**Affiliations:** ^1^ Department of Endocrinology, Guang’anmen Hospital, China Academy of Chinese Medical Sciences, Beijing, China; ^2^ Clinical Department of Traditional Chinese Medicine, Beijing University of Chinese Medicine, Beijing, China

**Keywords:** chinese patent medicines, efficacy, non-proliferative diabetic retinopathy, randomized controlled trials (RCT), calcium dobesilate

## Abstract

**Background:**

Diabetic retinopathy (DR), one of the commonest microvascular complications in diabetic patients, is featured by a series of fundus lesions. Conventional Western medicine therapies for DR are always with modest treatment outcome. This paper is to assess the ocular fundus signs, vision and safety of Chinese patent medicines (CPMs) as an add-on treatment for DR.

**Method:**

7 electronic databases were searched to determine eligible trials. Randomized controlled trials (RCTs) of non-proliferative diabetic retinopathy (NPDR) in which the intervention group received CPMs combined with calcium dobesilate (CD), and the control group received only CD were included for analysis. Two reviewers extracted the data independently. Results expressing as mean differences (MD) and relative risks (RR) were analyzed with a fixed-effects or random-effects models.

**Results:**

19 RCTs involved 1568 participants with 1622 eyes met our inclusion criteria. The results suggested that compared with CD alone, CPMs plus CD for NPDR was superior at reducing the microaneurysm volume (MD -3.37; 95% confidence interval [CI], -3.59 to -3.14), microaneurysm counts (MD -2.29; 95%CI -2.97 to -1.61), hemorrhage area (MD -0.79; 95%CI -0.83 to -0.75), and macular thickness (MD -59.72; 95%CI -63.24 to -56.20). Participants in CPMs plus CD group also achieved a better vision. No obvious adverse events occurred.

**Conclusion:**

CPMs as an add-on therapy for NPDR have additional benefits and be generally safe. This meta‐analysis demonstrated that CPMs combined with CD could improve retinal microaneurysm, hemorrhage, macular thickness, visual acuity, fasting blood glucose (FBG), and glycosylated hemoglobin (HbAlc) compared with CD alone. Further studies are needed to provide more conclusive evidence.

**Systematic Review Registration:**

PROSPERO https://www.crd.york.ac.uk/prospero/, identifier CRD42021257999.

## Highlights

1. CPMs as an add-on therapy showed clinically and statistically significant reductions in microaneurysm, hemorrhage, macular thickness, visual acuity, FBG, and HbAlc for NPDR.
2. The outcomes can evaluate the therapeutic effects of CPMs in the treatment of NPDR objectively, indicating that CPMs might be used as a complementary and alternative approach to prevent, delay and reverse DR progression.

## Introduction

In parallel with the soaring prevalence of diabetes mellitus (DM) to an epidemic proportion (1), the incidence of diabetic retinopathy (DR) is inevitably increasing. A meta-analysis that included 35 epidemiological studies around the world showed that the prevalence of DR was as high as 34.6% among diabetic patients, respectively (2). In China which is home to the largest number of people with DM in the world, DR incidence fluctuated dramatically by region between 7.4% and 43.1%, and after 10 to 20 years, the incidence will increase to 54% (3–6). DR, a common retinal microvascular complication of DM, is responsible for progressive vision impairment and ultimately blindness (7). The quality of life, psychological aspects, as well as social behavior are irreversibly and seriously affected in patients with sight-threatening retinopathy. Moreover, apart from diabetic medication, patients with DR also need fundus examination and treatment resulting in substantial social and economic burden, which increases with retinopathy severity and visual impairment (8, 9).

According to the international staging system, DR is mainly divided into non-proliferative diabetic retinopathy (NPDR) characterized by microaneurysms, retinal dot and blot hemorrhages, hard exudates or cotton wool spots and proliferative diabetic retinopathy (PDR) characterized by neovascularization, vitreous or preretinal hemorrhages identified in fundoscopic examination (10, 11). The main therapeutic strategies for NPDR involve control of risk factors and microcirculation improvement. In this stage, screening and early effective intervention can prevent or delay the occurrence of the disease and avoid severe loss of vision. While PDR is usually treated with anti-vascular endothelial growth factor (VEGF) agents, laser and surgery (10, 12). Calcium dobesilate (CD), registered in more than 20 countries, is an established vasoactive and angioprotective drug that has been prescribed as the routine medication for decades to patients with NPDR to ameliorate microcirculatory disturbance (12, 13). As detailed in the meta-analysis study published in 2015, CD can reduce the hyperviscosity of blood, inhibit the synthesis and release of platelet aggregator, and improve retinal microangioma and hemorrhage (14). However, some patients did not benefit from CD treatment alone (15).

From another perspective, as the most important part of traditional Chinese medicine (TCM), Chinese patent medicines (CPMs), widely and conveniently used in clinical practice by TCM or non-TCM persons, are always based on well‐established and long‐standing prescriptions to meet the demands of TCM for syndrome differentiation. Remarkable progress has been made toward the treatment of DM and its complications with CPMs (16–18). In recent years, several RCTs indicated that CPMs as an adjunct therapy can enhance the therapeutic efficacy (e.g. improving ocular fundus signs including microaneurysm, hemorrhage and macular thickness, and vision) of CD (19, 20). Nevertheless, for now, there has been no systematic review data available concerning the ocular fundus signs, vision and safety of CPMs in combination with CD in treating NPDR. Therefore, we conducted a meta-analysis to evaluate the ocular fundus signs, vision and safety of CPMs as an adjunct therapy for the treatment of NPDR to provide high-quality evidence to help clinicians select better treatment strategy.

## Methods

This study complied with the Preferred Reporting Project (PRISMA) (21) statement for systematic reviews and meta-analysis and was registered through PROSPERO (PROSPERO Registration number: CRD42021257999).

### Search Strategy

We comprehensively searched the following databases from their start date to the present (up to 30 May 2021) and updated the search on 29 January 2022 to obtain more up-to-date and comprehensive evidence: PubMed, Embase, Cochrane Library, Wanfang database, Weipu database, China National Knowledge Infrastructure, Chinese biomedical literature database and clinical trial registration centers, such as ChiCTR and clinical Trials.gov.

To fully retrieve eligible studies, a combination of Medical subject headings (MeSH) and free text words were used. English search terms include: (Diabetic Retinopathy OR Diabetic Retinopathies OR Retinopathies, Diabetic OR Retinopathy, Diabetic) AND [Calcium Dobesilate OR Dobesilate, Calcium OR Dobesilate Calcium OR Calcium, Dobesilate OR 2,5-Dihydroxybenzenesulfonate OR 2,5 Dihydroxybenzenesulfonate OR 2,5-Dihydroxybenzenesulfonic Acid OR 2,5 Dihydroxybenzenesulfonic Acid OR Doxium OR Calcium Dobesilate Monoammonium Salt OR Calcium Dobesilate Monopotassium Salt OR Dexium OR Dobica OR Calcium Dobesilate (1:1)] AND (danshen OR dan shen OR xueshuantong OR xue shuan tong OR qiming OR qi ming OR qijudihuang OR qi ju di huang OR shuangdanmingmu OR shuang dan ming mu). All titles and abstracts of articles were then separately screened by two authors (Yuehong Zhang, Xuedong An). Any discrepancies in extraction were resolved through discussion.

### Study Selection

#### The Inclusion Criteria Were as Follows

1. The study included the patients who suffered from NPDR in accordance with international or domestic diagnostic criteria.
2. CD in combination with CPMs was used as an intervention, and CD alone was used as a control. Both groups received basic treatment (eg, glycemic control, blood pressure control and lipid modulation).
3. The outcomes included microaneurysm, hemorrhage, macular thickness, visual acuity, FBG, HbAlc, as well as adverse events (AEs).
4. We merely included trials whose treatment duration continued for 12 weeks or more.
5. The study design was a two-arm, randomized controlled trial.

#### The Exclusion Criteria Were as Follows

1. Non-randomized controlled clinical trials.
2. Studies with a treatment duration of less than 12 weeks.
3. Duplicate publication, only abstract or lack of outcome data and no access to obtain the full text.

### Data Extraction

The relevant information was extracted independently by 2 authors (Yuehong Zhang, Xuedong An). The details included title, authors, year published, duration of disease, sample size, age, gender, outcome indicators, intervention and control, intervention time, and AEs. Discrepancies were settled by consensus or third-party adjudication (Fengmei Lian).

### Quality Assessment

Two authors (Yuehong Zhang, Xuedong An) independently evaluated risk of bias of each of all included RCTs using the Cochrane Bias Risk Tool (CRBT) (22). This tool includes random sequence generation, allocation concealment, blinding, incomplete outcome data, selective reporting, and other biases. The results of the assessment were expressed as low bias risk, high bias risk and unclear bias risk.

### Statistical Analysis

RevMan5.4 software provided by the Cochrane collaboration network was used for system evaluation and meta-analysis. The heterogeneity of the included studies was assessed using the I^2^ statistics. I^2^ < 50% indicates that heterogeneity is reasonable among the groups, thus the fixed-effect model is adopted for analysis; otherwise, a random-effects model will be applied. Mean differences (MDs) were employed in continuous variables. All effect quantities were reported with 95% CI, and P ≤0.05 was considered a significant statistical difference. We also examined potential publication bias by constructing a funnel plot according to the Cochrane Handbook.

## Results

**Figure 1:**
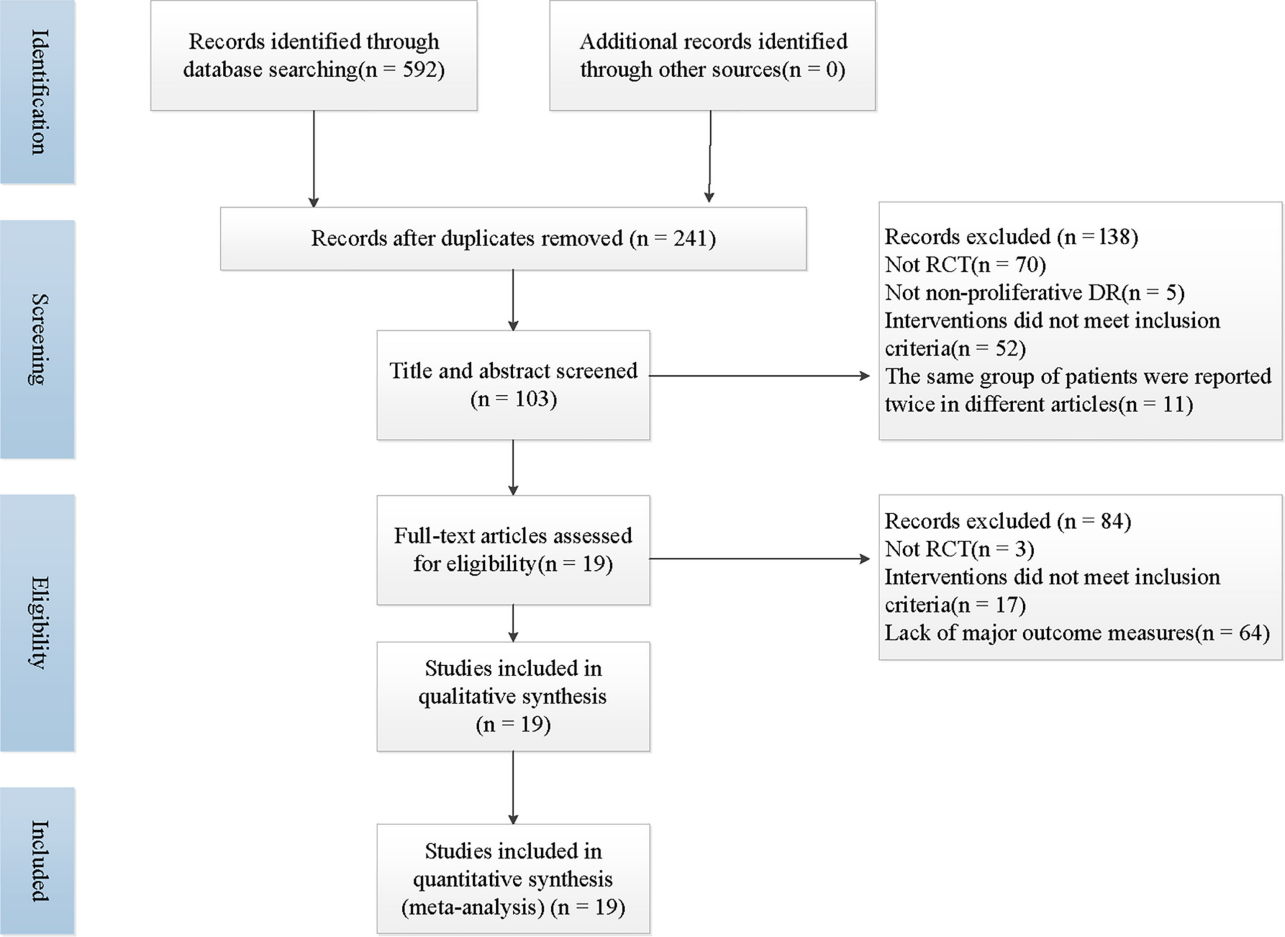
The screening process summarized in a flow diagram.

A total of 592 articles was retrieved by searching the literature databases, and *via* a search of clinical trial registries, we retrieved 5 records. After 351 duplicated studies were excluded, 241 studies remained. According to the understanding of the titles, abstracts, or summaries, we eliminated 138 articles that were not randomized, controlled trials, or the trial participants and the interventions did not meet our inclusion criteria. Then, after reading the full-text reading, 84 articles that full texts could not be retrieved, outcome data were missing, or the intervention did not match the criteria for integration strategies were deemed unsuitable and were therefore excluded. Finally, 19 trials were included in the present study (23–41). The specific literature screening and selection is shown in **Figure 1**.

A total of 1568 patients with 1622 eyes in the 19 RCTs met the inclusion criteria (23–41) (**Table 1**). The application of the treatment was assigned to two groups, control group (n=782, 809 eyes) that was treated with CD and intervention group (n=786, 813 eyes) that was treated with CPMs including Compound Xueshuantong (CX), Compound Danshen Dripping Pill (CDDP) and Shuangdan Mingmu Capsule (SMC) in combination with CD.

**Table 1:** Basic characteristics of included studies.

Study ID	Sample size (eyes)	Gender M/F; Age, yr		Disease course		Intervention time(month)	Main indicators	Method of random allocation
		Intervention group	Control group	Intervention group	Control group			
An 2020 (23)	70 (70)	19/16;51.17 ± 17.83	20/15;52.12 ± 15.76	3.71 ± 1.31, yr	3.51 ± 1.44, yr	3	A,D	Random grouping
Bai 2017 (24)	76(76)	20/18;62	21/17;51	N	N	4	A,C,D,E	Random number table method
Chai 2018 (25)	107(107)	32/22;61.11 ± 6.01	30/23;61.19 ± 6.03	2.77 ± 0.32, yr	2.79 ± 0.30, yr	3	B,C,D	Random grouping
Chen 2019 (26)	78(78)	21/18;59.34 ± 3.27	20/19;58.17 ± 3.82	2.24 ± 1.08, yr	2.38 ± 1.14, yr	3	A,C,D,E	Random grouping
Huang 2020 (27)	40(40)	9/11;53.16 ± 2.26	10/10;52.16 ± 2.45	N	N	4	A,C,D,E,F,G	Random grouping
Li 2018 (28)	60(60)	18/12;56.68 ± 2.52	17/13;55.72 ± 2.31	35.49 ± 1.04, mo	35.91 ± 1.23, mo	5	A,D	Random grouping
Liu 2019 (29)	120(120)	33/27;57.54 ± 8.11	32/28;57.10 ± 9.26	10.46 ± 8.16, yr	10.46 ± 8.16, yr	4	C,D	Random number table method
Ma 2018 (30)	54(68)	16(19)/11(15); 53.02 ± 4.13	15(20)/12(14);53.08 ± 4.25	3.51 ± 0.30, yr	3.67 ± 0.75, yr	5	A,C,D,E,F,G	Random number table method
Pei 2015 (31)	64(64)	17/15;56.4 ± 2.1	16/16;55.3 ± 1.2	35.8 ± 1.3, mo	40.2 ± 1.4, mo	5	A,C,D	Random grouping
Ruan 2017 (32)	70(70)	18/17;52.5 ± 1.1	20/15;52.8 ± 1.7	N	N	4	A,C,D,E,F,G	According to the order of visit
Wang 2020 (33)	86(86)	25/19;69.52 ± 7.11	23/19;68.35 ± 6.82	3.14 ± 1.45, yr	3.05 ± 1.32, yr	5	A,C,D	Random number table method
Wu 2018 (34)	72(92)	20(26)/16(20); 52.97 ± 4.17	20(26)/16(20); 52.97 ± 4.17	3.47 ± 0.81, yr	3.47 ± 0.81, yr	3	A,C,D,F,G	According to the order of visit
Xu 2019 (35)	86(86)	24/19;53.11 ± 4.41	25/18;53.06 ± 4.39	N	N	4	A,C,D	Random number table method
Yu 2017 (36)	68(68)	19/15;57.4 ± 8.3	17/17;58.1 ± 7.9	33.6 ± 2.7, mo	33.8 ± 2.6, mo	3	B,C,D	Random number table method
Zhang 2020a (37)	200(220)	60(68)/40(42); 53.86 ± 4.27	60(68)/40(42); 53.86 ± 4.27	3.48 ± 0.90, yr	3.48 ± 0.90, yr	12	A,C,D	Random grouping
Zhang 2020b (38)	80(80)	23/17;53.60 ± 5.20	24/16;53.30 ± 5.60	3.20 ± 1.60, yr	3.10 ± 1.50, yr	3	A,C,D	Random grouping
Zhao 2019 (39)	87(87)	22/22;53.66 ± 5.49	22/21;53.71 ± 5.52	N	N	6	A,C,D	Random grouping
Huang 2021a (40)	90(90)	28/17;67.5 ± 5.30	29/16;67.30 ± 5.10	2.60 ± 1.20	2.40 ± 1.10	6	A,G	Random number table method
Huang 2021b (41)	60(60)	17/13; 52.85 ± 6.38	18/12; 51.86 ± 6.16	3.21 ± 0.66	3.18 ± 0.72	5	A,C,D	Random number table method

A, Microaneurysm Volume; B, Microaneurysm counts; C, Hemorrhage area; D, Macular thickness; E, Visual acuity; F, FBG; G, HbAlc.

**Figure 2:**
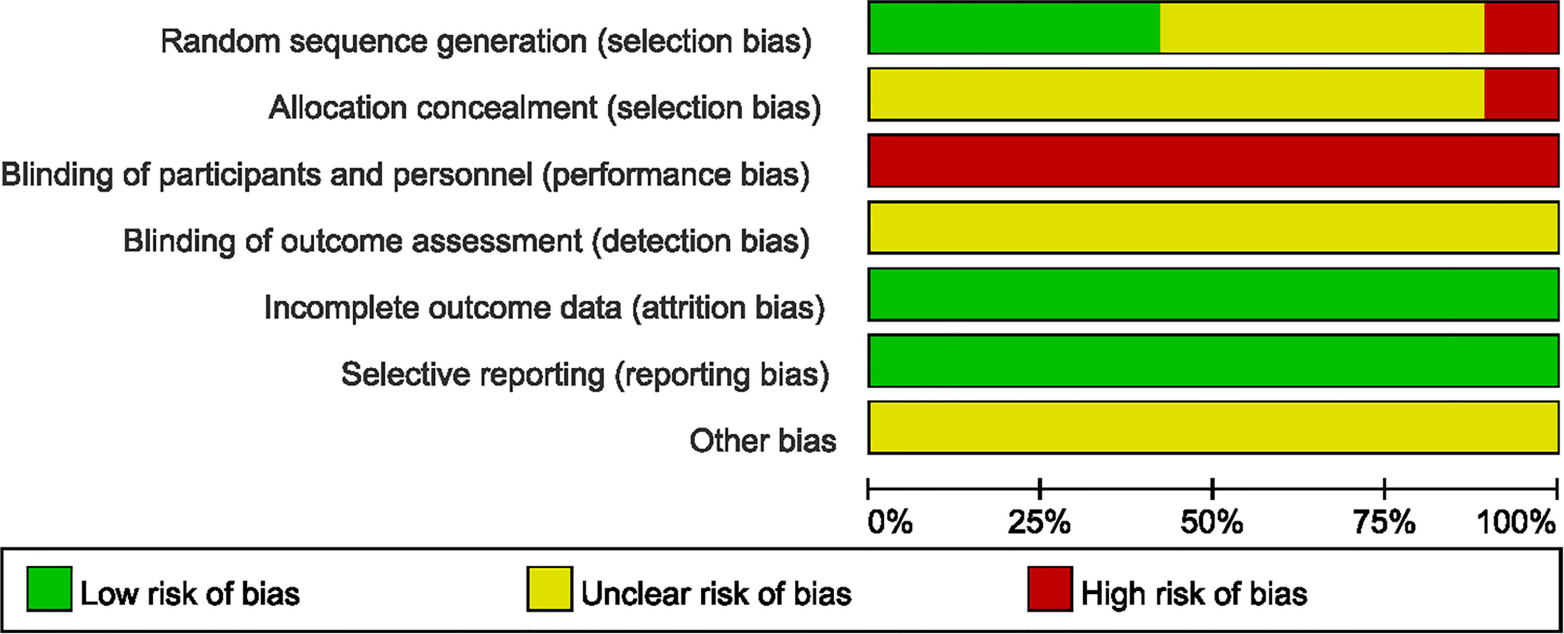
Quality assessment of the included trials-Risk of bias graph.

Hypoglycemic therapy was concomitantly administered to both groups to control glycemia. All 1568 patients were treated for at least 3 months. 8 studies (24, 29, 30, 33, 35, 36, 40, 41)mentioned the generation of random sequences (used random number tables), 2 RCTs (32, 34) used a semi-randomization method, and participants were assigned according to the visiting sequence, the remaining 9 RCTs (23, 25–28, 31, 37–39) referred to “random” but no method in detail. No study clearly mentioned the allocation concealment or the use of blind method, thus, all of them were judged to be at high or unclear risk of bias concerning the 2 items. None of the studies described the number of dropouts or lost to follow-up cases. We considered most studies to be at of low risk of reporting bias (**Figure 2**).

### Microaneurysm Volume

**Figure 3:**
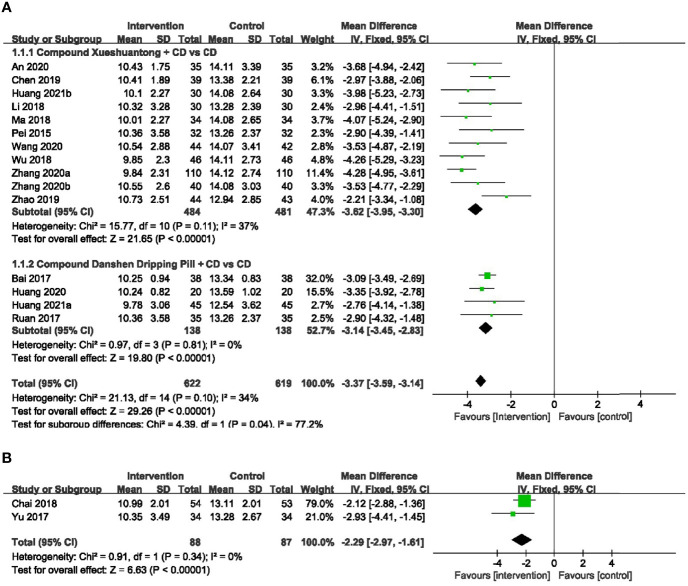
Forest plots of comparison of ocular fundus signs for CPMs plus CD versus CD alone. **(A)** Microaneurysm volume, **(B)** Microaneurysm counts.

A total of 15 RCTs (23, 24, 26–28, 30–34, 37–41) with 1241 eyes provided data for microaneurysm volume. There was no significant heterogeneity reported, and a fixed-effects model was used. Meta-analysis result showed that CPMs combined with CD was significantly superior to control group in reducing microaneurysm volume (n=1241, MD -3.37, 95% CI -3.59 to -3.14), and the difference had statistical significance (P < 0.00001). According to the different CPMs, subgroup analysis was performed. 11 studies (23, 26, 28, 30, 31, 33, 34, 37–39, 41) reported the information of CX concerning the reduction of microaneurysm volume. The results revealed that a statistically significant decrease in microaneurysm volume with CX plus CD, compared to the CD group (n=965, MD -3.62, 95% CI -3.95 to -3.30). There was also a significant difference between the subgroups of the CDDP plus CD and CD groups (24, 27, 32, 40) (n=276, MD -3.14, 95% CI -3.45 to -2.83) (**Figure 3A**).

### Microaneurysm Counts

2 RCTs (25, 36)reported the number of microaneurysm to be the outcome. A fixed-effects model was used to analyze the dependent variables according to the heterogeneity without significant difference. Pooled analysis showed a statistically significant decrease in microaneurysm counts with CMPs plus CD group, compared to the CD group (n=175, MD -2.29, 95%CI -2.97 to -1.61) (**Figure 3B**).

### Hemorrhage Area

**Figure 4:**
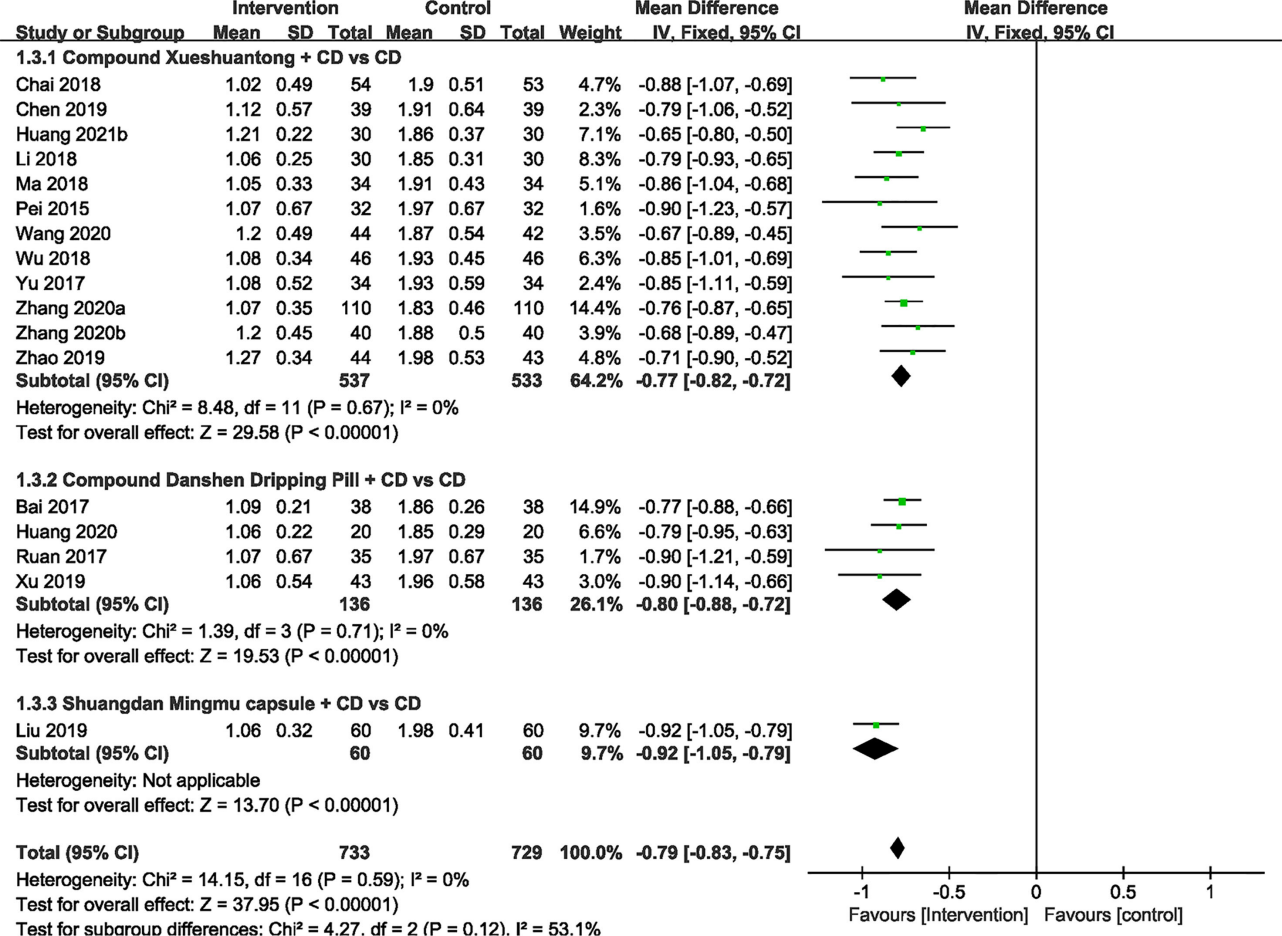
Forest plots of comparison of ocular fundus signs for CPMs plus CD versus CD alone. Hemorrhage area.

No significant heterogeneity was exhibited within 17 RCTs (24–39, 41) with available hemorrhage area data. Hence, we applied the fixed effect model. The results showed that hemorrhage area of CMPs in combination with CD in the treatment of NPDR was significantly smaller than that of CD alone (n=1462, MD -0.79, 95%CI -0.83 to -0.75). Subgroup analyses revealed that the pooled mean difference of hemorrhage area reduction was -0.77 mm^2^ (n=1070, 95%CI -0.82 to -0.72, P<0.00001) between CX plus CD and CD alone groups (25, 26, 28, 30, 31, 33, 34, 36–39, 41). In the CDDP combined with CD group versus the CD alone subgroup, 4 trials (24, 27, 32, 35) reported the hemorrhage area data. There was also a significant difference between the 2 groups (n=272, MD -0.80, 95% CI -0.88 to -0.72, P<0.00001). In 1 RCT (29) of SMC, results indicated that there was statistical difference (n=120, MD -0.92, 95% CI -1.05 to -0.79, P<0.00001) (**Figure 4**).

### Macular Thickness

**Figure 5:**
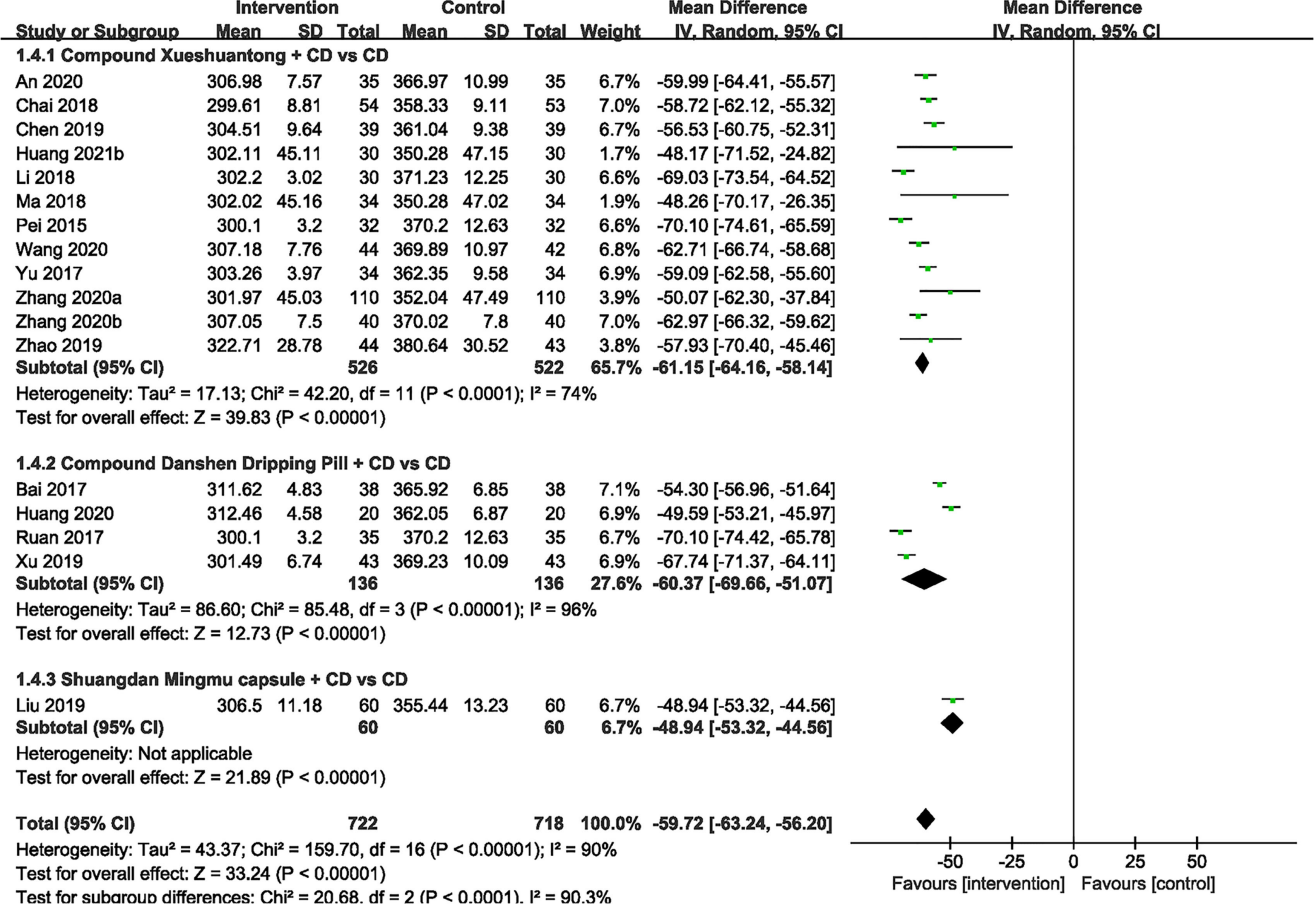
Forest plots of comparison of ocular fundus signs for CPMs plus CD versus CD alone. Macular thickness.

17 included studies (23–33, 35–39, 41)compared the effect of CPMs and CD on macular thickness. Significant heterogeneity was found (P < 0.00001, I^2^ = 90%), so a random effect model was used. The pooled mean differences of macular thickness reduction were -59.72μm (n=1440, 95% CI -63.24 to -56.20, P < 0.00001), and -61.15μm (n=1048, 95% CI -64.16 to -58.14, P < 0.00001), -60.37μm (n=272, 95% CI -69.66 to -51.07, P < 0.00001) and -48.94μm (n=120, 95% CI -53.32 to -44.56, P < 0.00001) for the CX plus CD and CD alone groups (23, 25, 26, 28, 30, 31, 33, 36–39, 41), the CDDP plus CD and CD alone groups (24, 27, 32, 35) and the SMC plus CD and CD alone groups (29), respectively (**Figure 5**).

### Visual Acuity

**Figure 6:**
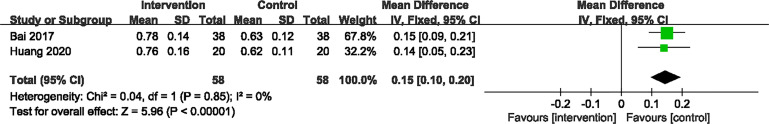
Forest plots of comparison of visual acuity for CPMs plus CD versus CD alone.

2 RCTs (24, 27) provided specific information concerning the improvement of visual acuity. Significant heterogeneity was not observed between these RCTs. A fixed-effect model revealed that the CPMs group was statistically different than the CD group in decreasing the visual acuity (n=116, MD 0.15, 95% CI 0.10 to 0.20, P < 0.00001) (**Figure 6**).

### FBG

**Figure 7:**
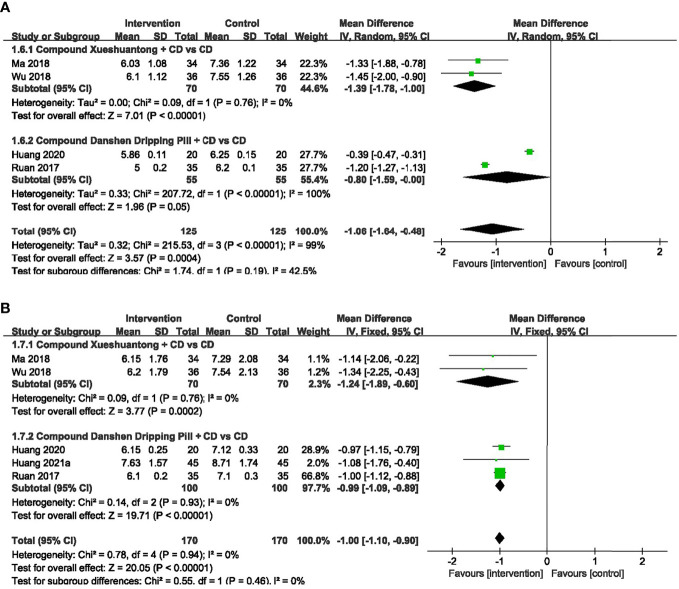
Forest plots of comparison of blood sugar for CPMs plus CD versus CD alone. **(A)** FBG, **(B)** HbAlc.

4 trials (27, 30, 32, 34) provided the data concerning the FBG. The pooled effect was generated using a random-effects model because of the significant heterogeneity. The results showed that FBG in CX combined group presented significantly lower than the CD group (30, 34) (n=140, MD -1.39, 95% CI -1.78 to -1.00, P<0.00001). Significant reduction was also found between the subgroups of the CDDP Combined group and CD group (27, 32) (n=110, MD -0.80, 95% CI -1.59 to -0.00, P<0.00001) (**Figure 7A**).

### HbAlc

5 trials (27, 30, 32, 34, 40) reported HbAlc data. As we found no heterogeneity among them, the fixed-effects model was selected. A pooled analysis of 2 trials (30, 34) showed a statistically significant decrease in HbAlc with CX combined group, compared to the CD group (n=140, MD -1.24, 95% CI -1.89 to -0.60, P=0.0002). HbAlc was also significantly lower in CDDP combined group versus CD group (27, 32, 40) (n=200, MD -0.99, 95% CI -1.09 to -0.89, P<0.00001) (**Figure 7B**).

### Adverse Events

**Figure 8:**
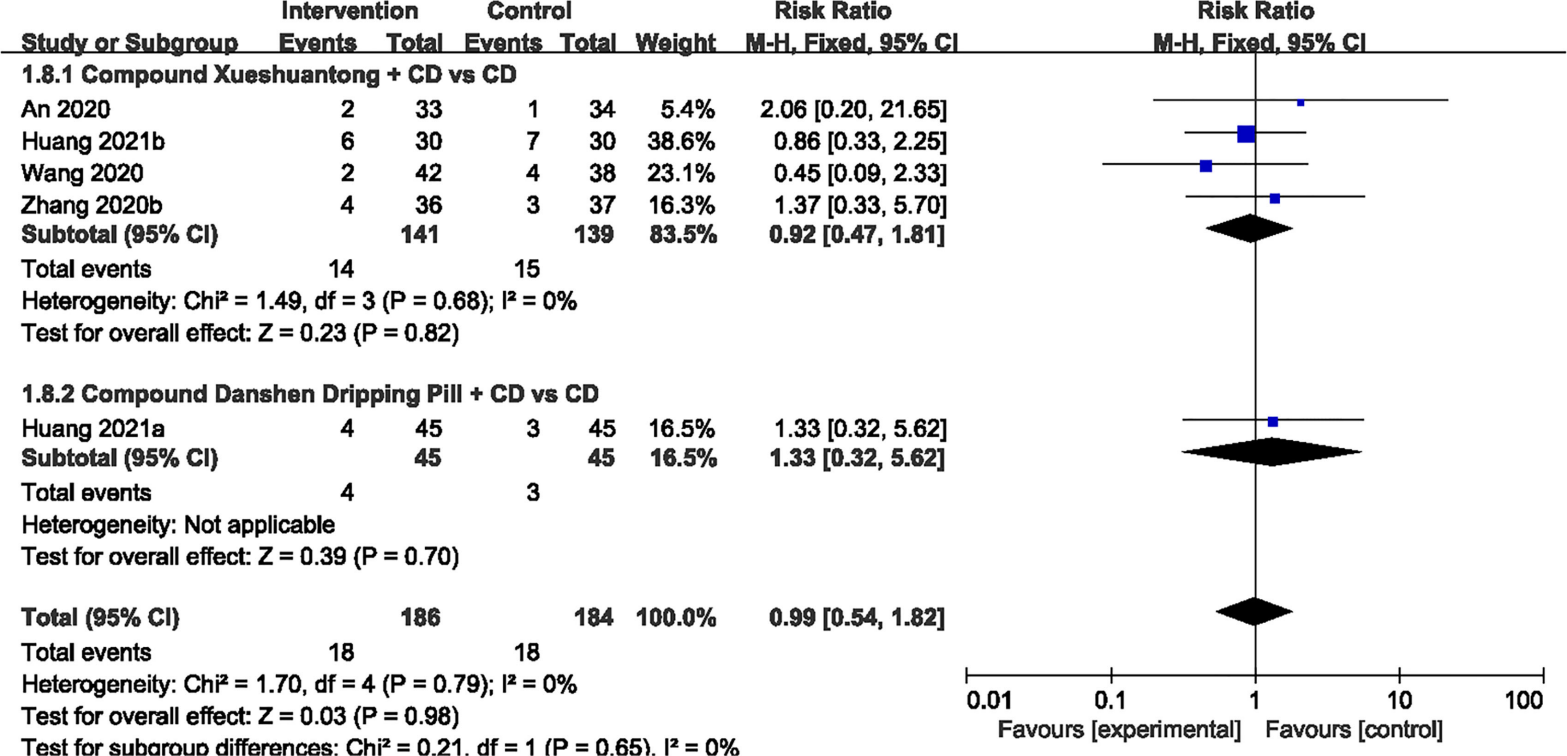
Forest plots of comparison of adverse events for CPMs plus CD versus CD alone.

Of the 19 studies (23–41), 6 reported no evident adverse reaction (24, 29–32, 35). 5 trials (23, 33, 38, 40, 41) showed that there was no significant difference in AEs between the CPMs plus CD and CD groups (n=370, RR 0.99, 95% CI 0.54 to 1.82) **Figure 8**. Adverse reactions in both groups were mainly concerned with stomach discomfort, nausea and decreased appetite.

### Publication Bias

**Figure 9:**
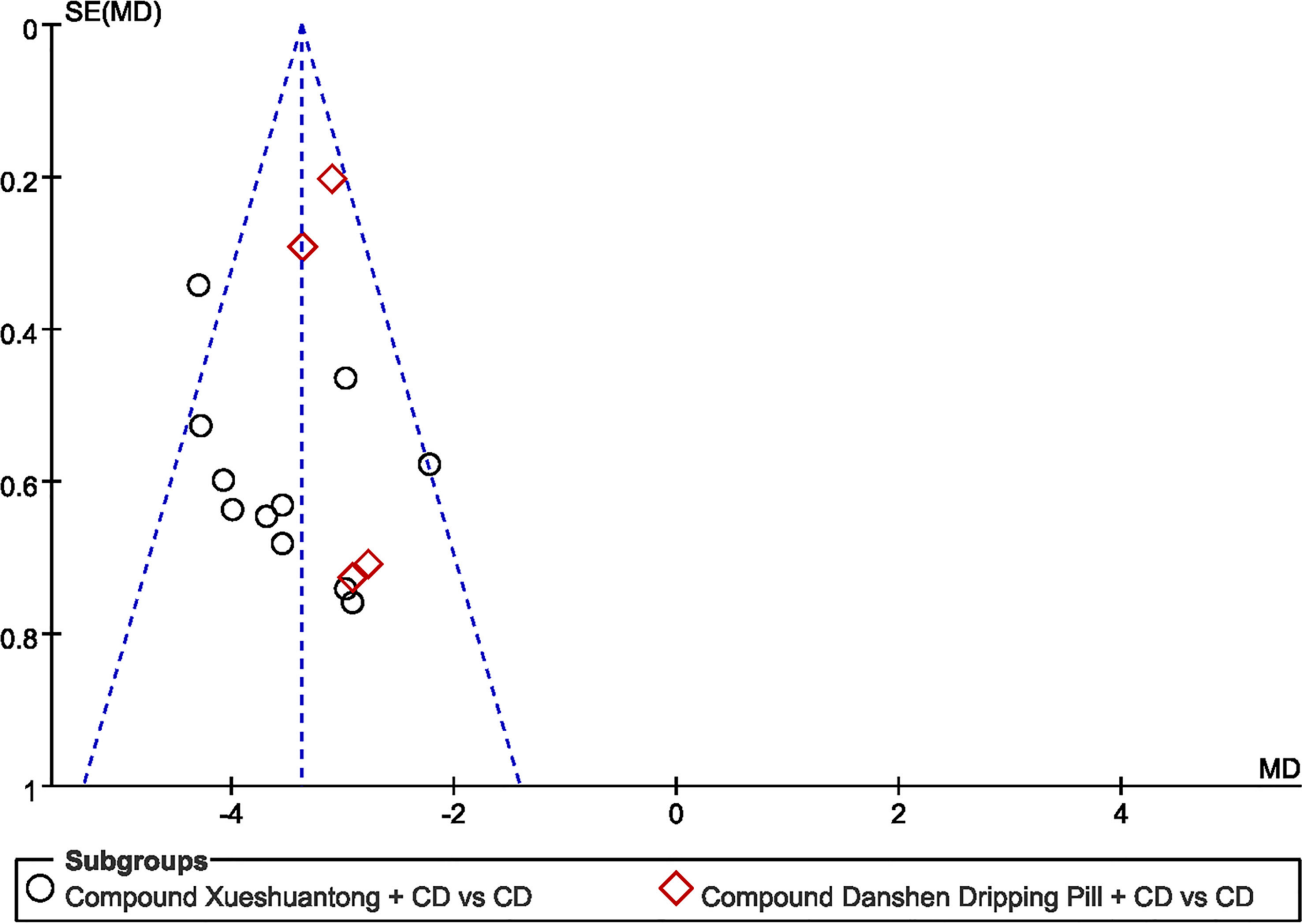
Funnel plot of the trials that compared CPMs plus CD group with CD group; Microaneurysm volume.

Funnel plots of microaneurysm volume was shown in **Figure 9**. Shape of the funnel plots was not completely symmetrical, suggesting probable publication bias. The following factors were linked with publication bias: positive results are easier to publish than negative results; the small sample size of the included studies brings a small sample effect.

## Discussion

Once patients have entered the stage of advanced DR, they are more susceptible to developing more severe forms of the disease such as macular edema, vitreous hemorrhage and retinal detachment. Even with surgical intervention, visual function suffers serious damage. Therefore, the early diagnosis of DR and adequate initiating therapy are essential, which remain a challenge (42). Telemedicine seems to be a worthy alternative, which can provide an easy, smart specialist fundus oculi examination, especially in times of pandemic, to bring the specialist closer to clinical centers allowing screening and follow-up of DR (43, 44). The other way round, screening for an early-stage diagnosis of DR allows not only to avoid the risk of severe vision loss but also to estimate high cardiovascular disease risk in diabetic patients (45), particularly if associated with additional important forms of diabetic microangiopathies, such as albuminuria (46).

As for therapy, the past decades have witnessed significant advances in the treatment options for DR. CD, as the most widely accepted oral vascular protective agent for NPDR, contributes a lot to the successful management in arresting or reversing the progression of the disease. The possible protective mechanisms of CD responsible for DR are mainly achieved by decreasing retinal albumin leakage and capillary permeability, suppressing oxidative stress, inhibiting aldose reductase (47–49). However, in parallel, TCM, as an adjuvant therapy option has gotten more and more attention among the public for its remarkable effects in clinical practice. It states that the occurrence of diseases is due to the imbalance of Yin, Yang, Qi and Blood, and CPMs are prescribed accordingly to rebuild that balance.

**Figure 10:**
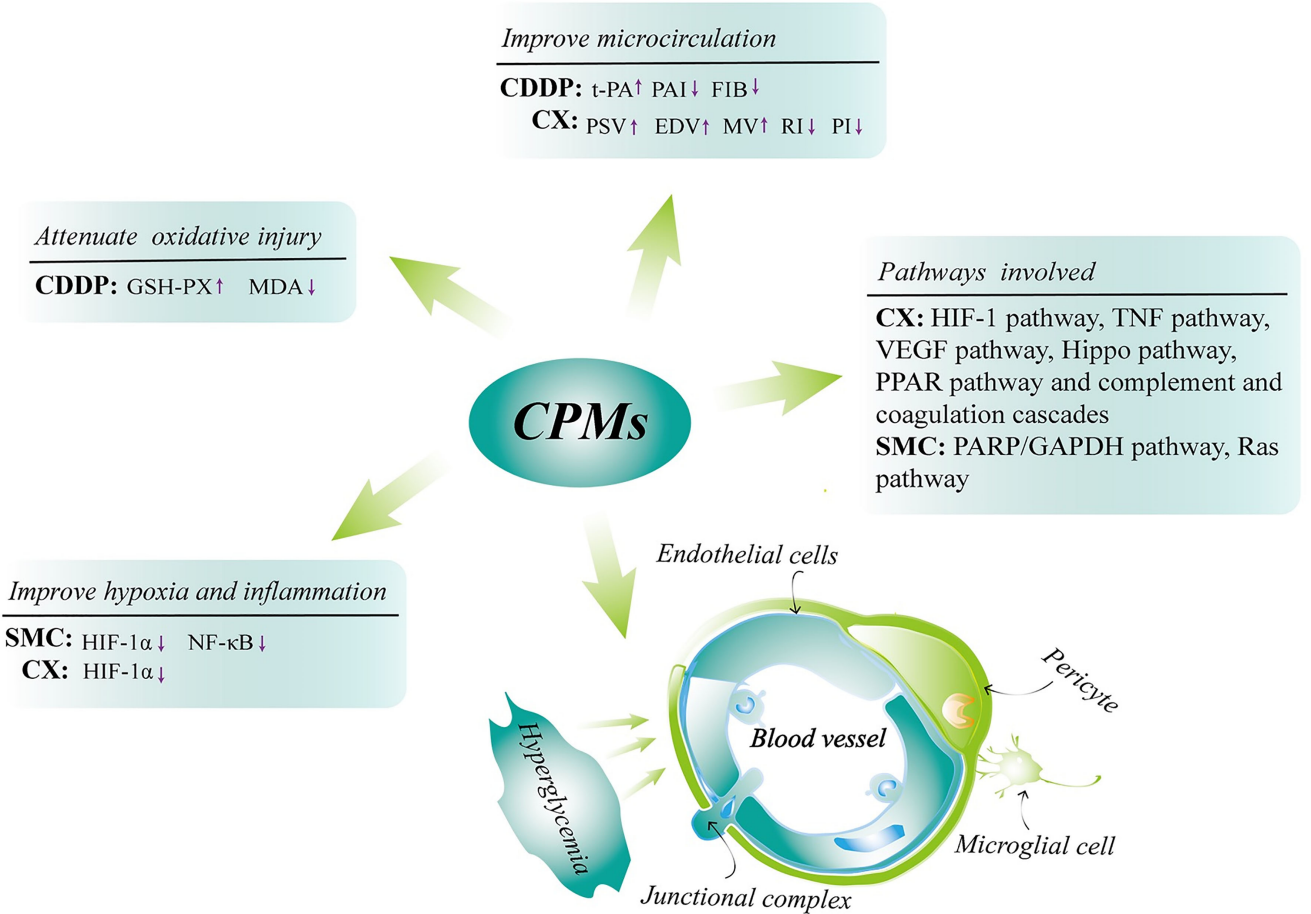
The major underlying mechanisms involved in the treatment of DR with CPMs (CX, CDDP and SMC). Chinese patent medicines (CPMs); Compound Xueshuantong (CX); Compound Danshen Dripping Pill (CDDP); Shuangdan Mingmu Capsule (SMC); tissue plasminogen activator (t-PA); plasminogen activator inhibitor (PAI); fibrinogen (FIB); peak systolic velocity (PSV); end diastolic velocity (EDV); mean velocity (MV); resistance index (RI); pulsatility index (PI); glutathione peroxidase (GSH-PX); malondialdehyde (MDA); hypoxia-inducible factor 1α (HIF-1α); nuclear factor-κB (NF‐κB).

Previous studies have proven that CPMs are a safe and effective therapeutic option in NPDR treatment (18, 50, 51). Our research team has completed a controlled trial enrolled with 223 NPDR patients indicating that significant effect was observed in CDDP (composed of three herbs: *Salvia miltiorrhiza, Panax notoginseng* and *Borneol*) treated group (18). We find that at 24 weeks, for the fluorescein fundus angiography, the percent of “Excellent” and “Effective” in the high-dose and mid-dose CDDP groups (540mg, 3 times/day) was 74% and 77%, respectively, that is significantly higher than 28% in the placebo group (P<0.001) (18). CDDP may exert its action mainly by improving retinal microcirculation and alleviating retinal tissue ischemia observed in an animal study (52). In the meantime, the research has also revealed that CDDP improved retinal damage in diabetic rats through attenuating oxidative injury (52). CX with daily doses used in therapy range from 1.5g up to 4.5g per day consists of four herbs including *Panax notoginseng, Salvia miltiorrhiza, Astragalus membranaceus* and *Scrophulariae Radix*. It counteracts DR may be related to the HIF-1 pathway, TNF pathway, VEGF pathway and Hippo pathway (53–55), thereby improving retinal hypoxic microenvironment, retinal inflammation and vascular proliferation. On the other hand, CX causes the hemodynamics and morphological alterations in diabetic rat retinas, which may *via* regulating the PPAR pathway and complement and coagulation cascades (56). SMC based on Chinese ancient prescriptions named Liuwei Dihuang Pill and Erzhi Pill is composed of *Ligustrum lucidum Ait., Eclipta prostrata, Cornus officinalis Sieb, Dioscorea polystachya Turczaninow, Salvia miltiorrhiza Bunge, Panax notoginseng, Paeonia suffruticosa Andr., Alismatis Rhizoma, Poria cocos (Schw.), Smilax glabra Roxb.* and *Achyranthes bidentata Blume*. The usual dose of SMC is a 2 g single dose three times daily for 4 months. As to the underlying mechanisms of SMC in the treatment of DR may be bound up with the down-regulation of the expressions of hypoxia-inducible factor 1α (HIF-1α) and nuclear factor‐βK (NF‐κB), as well as an attenuation of oxidative stress-induced apoptosis of pericytes through PARP/GAPDH pathway and inhibition of retinal angiogenesis *via* Ras pathway consequently exerting retinal tissue-protective effects (57–59). The major mechanisms involved in the treatment of DR with CPMs mentioned above are shown in **Figure 10**. Additionally, it was shown that all these CPMs contained *Salvia miltiorrhiza* and *Panax notoginseng*, so the information can be used to support further single herb studies.

### Effectiveness and Safety of CPMs Combined With CD for NPDR

The aim of this meta-analysis was to assess the ocular fundus signs, vision and safety of CPMs as an adjuvant therapy for DR. 19 studies (23–41)with 1622 eyes meet the criterion. The results demonstrated that CPMs provided additional benefits for the NPDR population, which displayed a significant improvement in ocular fundus signs including microaneurysm volume/counts, hemorrhage area and macular thickness as well as visual acuity, FBG and HbAlc compared to CD alone. The effects of the combination of CPMs and CD may be related to their mechanisms of the synergistic effect between the two drugs, which remain to be further studied.

The data to the analyses on treatment-associated AEs was provided in 5 (23, 33, 38, 40, 41) out of 19 studies (23–41). Stomach discomfort, nausea and appetite loss were the manly common AEs. Meta-analysis results were basically consistent with relevant study results with no significant differences between the two groups, demonstrating that a combination of CPMs and CD is safe for the treatment of DR. However, due to lack of long-term follow-up data, long-term efficacy and safety could not be determined. Thus, the safety of the combination with CPMs and CD still needs to be observed.

### Description of the Outcomes

NPDR, the early stage of DR, is often asymptomatic and mostly accompanied by microaneurysms and hemorrhages as the ocular fundus signs (60). Microaneurysms, the signature lesion of NPDR, appear as dilations of capillaries on the fundus that is widely performed to measure the severity of DR and assess the risk of future DR progression (61). Hemorrhage resulting from the rupture of microaneurysms is another essential diagnostic criterion to assess the severity level of DR (11). Therefore, microaneurysms and hemorrhage can be used as suitable indicators to evaluate drug effects. The macula is particularly prone to fluid accumulation leading to macular edema which is the abnormal thickening of the macula (62). This thickening always threatens or actually causes vision loss. Thus, it is particularly important to evaluate macular thickness. The main causative pathogenic factor in the progression of DR is hyperglycemia, and better glycemic control may be beneficial in reducing the incidence of DR and increasing the odds of improvement of DR (14). In our study, CMPs as an add-on therapy for NPDR could better control the FBG and HbAlc of the subjects. However, significant heterogeneity remained in the CDDP subgroups under the heading of FPG, several potential causes could be associated with the severity of disease and different hypoglycemic agents with different pharmacokinetic parameters.

### Potential Limitations of the Study

Several limitations of our study have to be acknowledged. First, none of the 19 articles (23–41) provided a complete trial protocol, making the data questionable in terms of standardization and transparency. Second, only 8 literature (24, 29, 30, 33, 35, 36, 40, 41) reported that participants were randomized using table of random numbers, more than half of the studies did not state the randomization process, which exposed the results to selection bias. Third, none of the 19 studies (23–41) mentioned how allocation concealment or blinding was performed, and participants withdraw and drop out, which may result in selection bias, information bias and publication bias. Fourth, the included populations were all Asian populations, and no studies of CPMs on other races were retrieved, thus the efficacy of CPMs combined with CD on NPDR in other races is unknown. Since the concern in methodological quality, multicenter, large-sample, well‐designed RCTs following the Consolidated Standards of Reporting Trials (CONSORT) guidelines (63) are needed.

## Conclusion

In summary, combination therapies hold great potential for NPDR. Compared with the use of CD alone, CPMs (CX, CDDP OR SMC) plus CD has additional benefits in the treatment of NPDR, especially in ocular fundus signs involving microaneurysm, hemorrhage and macular thickness, and visual acuity. It also had advantages in the reduction of FBG and HbAlc without serious adverse reactions. These effects largely arrest progression of DR and save vision efficaciously. However, due to the moderate study quality and possible publication bias, the results should be treated with caution. In the future, large-scale RCTs with powerful study design are desired to provide a higher grade of evidence.

## Data Availability Statement

The original contributions presented in the study are included in the article/supplementary material. Further inquiries can be directed to the corresponding author.

## Author Contributions 

FL designed the study and as the corresponding author. DJ, YD, and RZ carried out the literature search. YueZ and XA contributed to data extraction and quality assessment and drafted the manuscript with LD. YuqZ and XK provided statistical supports for meta-analysis. All authors contributed to the article and approved the submitted version.

## Funding

This work was supported by 2015 Traditional Chinese Medicine Scientific Research (201507001-11) and Innovation Team and Talents Cultivation Program of National Administration of Traditional Chinese Medicine (No: ZYYCXTD-D-202001). The funders had no role in the study design, data collection, data analysis, interpretation, or writing of the report.

## Conflict of Interest

The authors declare that the research was conducted in the absence of any commercial or financial relationships that could be construed as a potential conflict of interest.

## Publisher’s Note

All claims expressed in this article are solely those of the authors and do not necessarily represent those of their affiliated organizations, or those of the publisher, the editors and the reviewers. Any product that may be evaluated in this article, or claim that may be made by its manufacturer, is not guaranteed or endorsed by the publisher.
